# Structural and Functional Impact of the G340S Mutation in Plasmodium falciparum Ferredoxin NADP⁺ Reductase: In silico Analysis and Molecular Docking

**DOI:** 10.12688/gatesopenres.16393.1

**Published:** 2026-07-30

**Authors:** Daniel Kiboi, Brenda Muriithi

**Affiliations:** 1 Jomo Kenyatta University of Agriculture and Technology, 62000, Nairobi, 00200, Kenya

**Keywords:** Plasmodium falciparum, Ferredoxin NADP+ Reductase, G340S mutation, Lumefantrine resistance, Molecular docking

## Abstract

*Plasmodium falciparum*, the deadliest malaria parasite, continues to burden health systems across sub-Saharan Africa, where artemether-lumefantrine remains a frontline treatment. Partial artemisinin resistance is emerging, increasing selection pressure on lumefantrine (LM), yet the molecular basis of reduced LM susceptibility remains poorly defined. While LM has been described as refractory to resistance, its efficacy could be compromised if resistance arises. To explore candidate determinants of LM response and guided by a Gly332Ser substitution previously identified in Ferredoxin NADP ^+^ reductase from LM-selected *Plasmodium berghei*, we investigated the homologous G340S variant in *Plasmodium falciparum* ferredoxin NADP ^+^ reductase ( *PfFNR*), a central enzyme of the apicoplast redox system. To predict the structural and functional impact of the G340S substitution, this study integrated sequence conservation analysis, AlphaFold 3 structural modelling, structural superimposition, molecular dynamics simulation, NADPH and NADP ^+^ docking, HADDOCK docking with *P. falciparum* ferredoxin ( *PfFd*), and comparative docking of lumefantrine, primaquine, artemisinin and dihydroartemisinin. Gly340 was conserved across the Plasmodium FNR orthologs examined and mapped to a constrained loop adjacent to the NADP ^+^ binding region. Structural superimposition showed that G340S preserved the global *PfFNR* fold but introduced localized changes near residue 340. Exploratory single-trajectory molecular dynamics suggests that G340S may alter PfFNR conformational sampling, with increased RMSD, radius of gyration, solvent-accessible surface area and residue-level flexibility. Cofactor docking predicted a modest mutation-associated shift, strongest for NADPH, whereas NADP ^+^ docking remained largely stable. HADDOCK analysis indicated altered *PfFNR-PfFd* docking preferences, and antimalarial docking showed ligand-dependent effects, greatest for artemisinin and dihydroartemisinin, with minimal lumefantrine change. Together, these data support G340S as a redox-associated candidate variant that may reshape cofactor and partner-interface, but not as a validated lumefantrine-resistance marker until confirmed by biochemical, genetic and parasite-based assays. The findings define testable mechanisms for future functional assays of *PfFNR* in antimalarial response phenotypes.

## Introduction

*Plasmodium falciparum* remains the leading cause of malaria mortality worldwide and continues to impose its greatest burden on children under five years of age and pregnant women in sub-Saharan Africa ( Ward et al., 2022; WHO, 2025). Despite continued gains from vector control and other preventive interventions, effective chemotherapy remains essential for clearance of parasite infections. Over the past two decades, artemisinin-based combination therapies (ACTs) have substantially reduced malaria morbidity and mortality and remain the cornerstone of treatment for uncomplicated *P. falciparum* malaria ( Eastman & Fidock, 2009; Rosenthal et al., 2024). Nevertheless, the global malaria burden remains high. According to the World Health Organization, malaria caused an estimated 610,000 deaths in 2024, with sub-Saharan Africa accounting for 95% of these deaths ( WHO, 2025), underscoring the continued dependence of malaria control on the efficacy of frontline antimalarial drugs. Currently, Artemether-lumefantrine (AL) remains one of the most widely used ACTs in Africa and continues to serve as first-line therapy in many endemic settings ( Rosenthal et al., 2024; WHO, 2022). However, the emergence and spread of parasite populations with reduced susceptibility to artemisinin and its long-acting partner drugs threaten the durability of ACT efficacy ( Balikagala et al., 2021; Dondorp et al., 2009). This concern is particularly relevant for lumefantrine (LM), which persists after the artemisinin component has largely been cleared and is therefore responsible for eliminating residual parasites and preventing recrudescence. While this pharmacological design underpins the success of AL, it also places lumefantrine under substantial post-treatment selective pressure. Reports of reduced susceptibility to both dihydroartemisinin and lumefantrine, together with therapeutic efficacy data and observations from imported malaria cases, suggest that AL performance may be under increasing strain in some African settings ( Mbye et al., 2022; Tumwebaze et al., 2022).

In contrast to artemisinin resistance, the molecular basis of reduced lumefantrine susceptibility in *P. falciparum* remains much less clearly defined. Associations have been described with polymorphisms in genes such as *pfmdr1*, particularly alleles including N86 and 184F, as well as variation in *pfmdr1* copy number ( Mungthin et al., 2010; Sisowath et al., 2005). Consistent with this, 2021 isolates from northern Uganda carrying the *P. falciparum* K13 469Y or 675V mutation were significantly less susceptible to lumefantrine than isolates from eastern Uganda ( Tumwebaze et al., 2022). However, robust, validated molecular markers and clearly defined mechanisms of lumefantrine resistance in *P. falciparum* remain elusive. This unresolved gap is important because AL is used at a massive scale across sub-Saharan Africa, and continued reliance on lumefantrine without a clearer understanding of resistance mechanisms could compromise both current treatment efficacy and the rational development of future regimens incorporating LM ( de Koning-Ward et al., 2021; Halsey & Plucinski, 2023). In the absence of stable LM-resistant *P. falciparum* isolates ( de Koning-Ward et al., 2021), one way to identify biologically plausible determinants of lumefantrine response is to examine loci that emerge under lumefantrine selection in experimental malaria systems. In our previous work, using the rodent malaria model *Plasmodium berghei* ANKA, we selected stable lumefantrine-resistant parasites, characterized their resistance phenotype, and identified a novel Gly332Ser substitution in the FNR gene ( Kiboi et al., 2014, 2020). Preliminary validation using an *fnr*-specific PlasmoGEM tagging vector further suggested that this substitution modified responses to lumefantrine and artemisinin in the rodent model. The homologous amino acid residue in *P. falciparum* is Gly340 in *PfFNR* ( Kiboi et al., 2020). Because the FNR locus was selected under lumefantrine pressure in a malaria model, the homologous residue in *PfFNR* becomes a biologically plausible site to examine in *P. falciparum.* This earlier *P. berghei* finding provides a useful starting point, but it does not by itself establish a lumefantrine-response mechanism in *P. falciparum*; here, we address that question at the level of structural and mechanistic inference.

*PfFNR* and its ferredoxin partner form the central redox pathway in the apicoplast, an organelle essential for parasite survival and for biosynthetic processes including fatty acid, heme, iron-sulfur cluster, and isoprenoid metabolism ( Ralph et al., 2004; Seeber & Soldati-Favre, 2010). Because of this central role, the *PfFNR/PfFd* system has long been considered a plausible antimalarial target ( Cichocki et al., 2021; Goodman & McFadden, 2013). More recent work has linked this pathway to parasite responses to redox-active compounds, including quinones, nitroaromatics, aromatic N-oxides, and artemisinin derivatives ( Lesanavičius et al., 2020). *PfFNR* is not only an apicoplast electron-transfer enzyme, but part of a redox system that can participate in redox cycling of antimalarial or xenobiotic compounds. Earlier work demonstrated FNR-dependent redox cycling of hydroxylated primaquine metabolites ( Vásquez-Vivar & Augusto, 1992), and more recent work showed *PfFNR*-catalysed redox cycling of plasmodione, supporting a broader role in parasite redox handling beyond basal metabolism ( Cichocki et al., 2021). *In vitro* studies have also shown that dihydroartemisinin partially inhibits *PfFNR* diaphorase activity by reducing its affinity for NADPH while preserving electron transfer to ferredoxin ( Kimata-Ariga et al., 2019; Kimata-Ariga & Morihisa, 2022). In addition, variation in *PfFd*, including the D97Y substitution that enhances binding to *PfFNR* and the Asp139Tyr mutation associated with artemisinin resistance in Southeast Asia, suggests that changes in the *PfFNR/PfFd* system may influence parasite redox balance under drug pressure ( Miotto et al., 2015). Collectively, these observations support the view that variation in the *PfFNR/PfFd* pathway could modify parasite responses to drug-induced redox stress; however, whether this system contributes to lumefantrine-response phenotypes in *P. falciparum* remains unknown. This unresolved question defines the central gap addressed in the present study.

Here, we investigated the homologous G340S variant in *P. falciparum FNR* based on the premise that, if Gly340 occupies a conserved and functionally important position, replacement with serine could reshape local structure, conformational dynamics, cofactor accommodation, and ferredoxin interaction in ways relevant to responses to lumefantrine, artemisinin, and primaquine. Because experimentally resolved mutation-specific *PfFNR* structures are not available, a comparative structural modeling framework was needed to evaluate how the G340S substitution might alter cofactor recognition, partner docking, and ligand accommodation. To test this idea, we performed sequence conservation analysis, AlphaFold 3-based structural modelling, structural superimposition, molecular dynamics simulations, nicotinamide cofactor docking, *PfFd-PfFNR* protein-protein docking, and comparative antimalarial docking to define the potential structural and functional consequences of the G340S substitution. We show that G340 is highly conserved across *Plasmodium FNR* orthologs and that substitution with serine does not disrupt the overall *PfFNR* fold. Instead, G340S induces localized remodeling near the cofactor-associated region, increases conformational flexibility, modestly alters predicted cofactor recognition, shifts *PfFd* docking preference, and produces ligand-dependent effects within the FAD-proximal pocket. These findings provide a structural framework for future laboratory experimental studies testing whether variation at the *PfFNR* locus contributes to lumefantrine-response phenotypes in *P. falciparum.*

## Materials and methods

**Figure 1.**
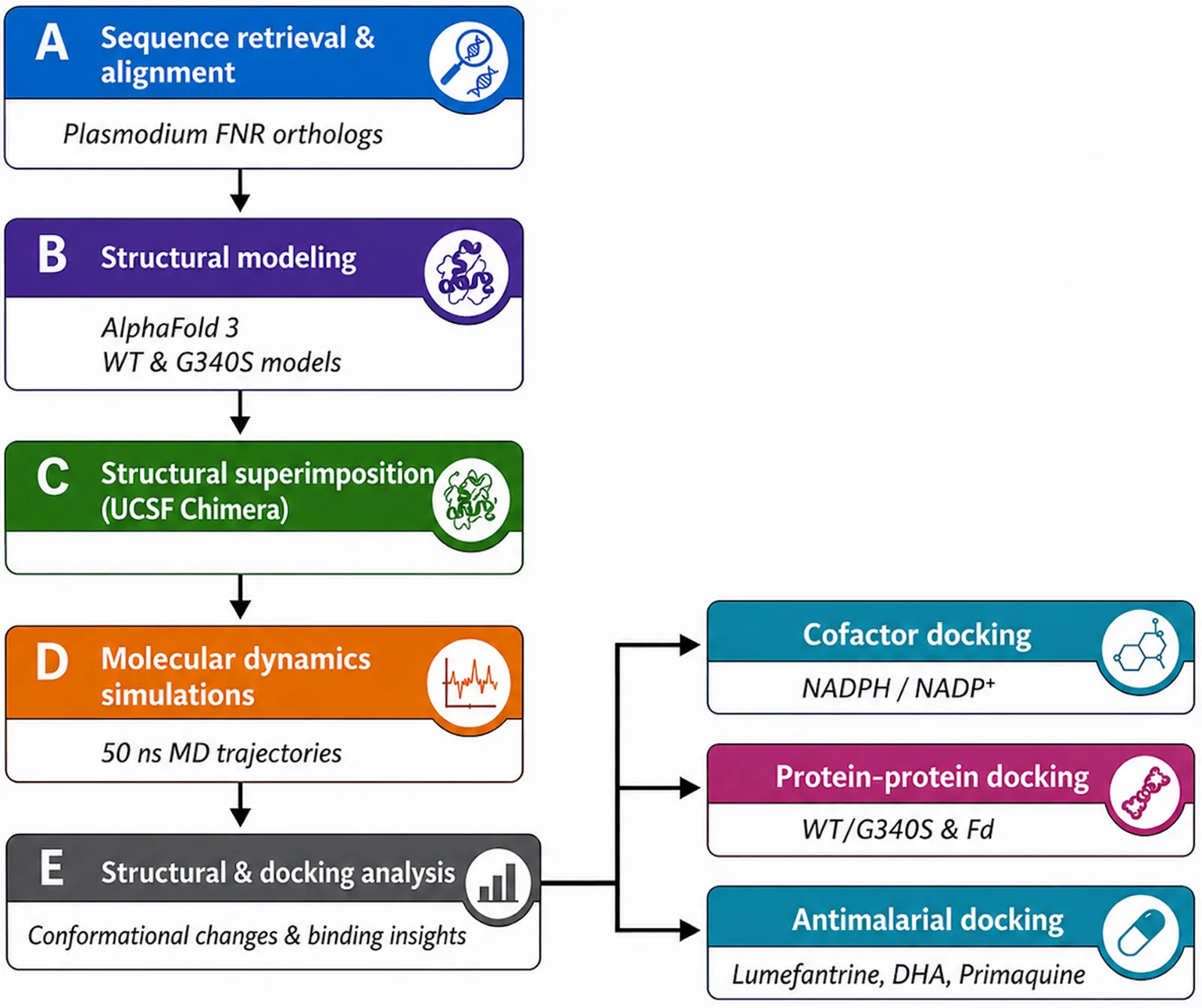
Overview of the computational workflow used to investigate the structural and functional consequences of the G340S substitution in *Plasmodium falciparum* ferredoxin NADP ^+^ reductase.

A multi-step computational workflow was used to investigate the structural consequences of the G340S substitution in *PfFNR.* As outlined in Figure 1, the workflow combined sequence retrieval and alignment, AlphaFold 3-based modelling of wild-type (WT) and G340S, structural superimposition, molecular dynamics simulations, and integrated structural and docking analyses.

### Sequence retrieval, conservation analysis, and domain annotation

Amino acid sequences of ferredoxin NADP ^+^ reductase (FNR) from *Plasmodium falciparum* 3D7 (PF3D7_0623200) and *Plasmodium berghei* ANKA (PBANKA_1122100), together with homologous sequences from *Plasmodium vivax* P01, *Plasmodium reichenowi* G01, and *Plasmodium adleri* L01, were retrieved from PlasmoDB ( https://plasmodb.org). Multiple sequence alignment was performed using Clustal Omega version 1.2.4 ( Sievers et al., 2011) to assess conservation across orthologs, with particular attention to *PfFNR* residue Gly340 and the corresponding Gly332 position in *PbFNR* ( Kiboi et al., 2020). To place the substituted residue within its structural context, the *PfFNR* sequence was further examined for conserved motifs and domains using MotifFinder together with Pfam v37.4 ( Mistry et al., 2020; Punta et al., 2011), PROSITE ( Sigrist et al., 2012), and the NCBI Conserved Domain Database (CDD) ( NCBI CDD). Attention was given to the Rossmann-like nucleotide-binding motif (Gly-x-Gly-x-x-Gly) in the N-terminal region and to acidic surface features implicated in ferredoxin docking.

### Structural modelling of WT and G340S *PfFNR*

To examine the structural consequences of the G340S substitution and generate receptor models for downstream docking analyses, structural models of WT and G340S *PfFNR* were generated by independent sequence-based prediction using the AlphaFold Server powered by AlphaFold 3 ( Abramson et al., 2024). The full-length WT *PfFNR* amino acid sequence was submitted directly, whereas the mutant sequence was generated by replacing glycine with serine at position 340 before separate submission. Thus, both WT and G340S structures were predicted *de novo* from sequence rather than generated by side-chain mutagenesis of a pre-existing WT model. Predicted structures were downloaded in CIF format and converted to PDB format for compatibility with downstream visualization, structural superimposition, molecular docking, and molecular dynamics workflows. Model confidence was assessed using the confidence metrics provided by the server, and the resulting WT and G340S models were retained for comparative structural analysis. For cofactor-aware structural comparison and all docking analyses, FAD-retained receptor models were generated by aligning the AlphaFold 3-predicted WT and G340S backbones to an FAD-bound *PfFNR* template and transferring the local FAD-containing cofactor environment to the corresponding predicted structures. The resulting WT and G340S receptor models were inspected to confirm preservation of the residue-340 environment and flavin-associated local architecture, and these FAD-bound models were retained as the principal receptor state for downstream structural comparison and docking analyses.

### Structural superimposition of WT and G340S *PfFNR*

To evaluate whether the G340S substitution altered the global architecture of *PfFNR*, structural superimposition of the WT *PfFNR* and G340S mutant models was performed using the Match → Align tool in UCSF Chimera v1.16 ( Meng et al., 2006). The G340S mutant model was superimposed onto the WT structure using corresponding C-alpha atom coordinates, with a residue–residue distance cutoff of 5.0 Å. The alignment was based on the spatial correspondence of structurally equivalent residues rather than sequence identity alone. Iterative refinement was performed over three cycles to improve the structural fit and exclude poorly aligned outlier regions. Structural similarity between the WT and G340S models was assessed using root mean square deviation (RMSD), pruned RMSD, Q-score, and structural distance measurement (SDM). RMSD was used to estimate the average positional deviation between aligned Cα atoms, while pruned RMSD was calculated after exclusion of poorly fitting residues to better represent the conserved structural core. The Q-score was used as a normalized measure of structural similarity, with values closer to 1 indicating greater correspondence between the aligned structures. SDM was used as an additional distance-based metric to evaluate structural divergence between the WT and mutant models.

### Molecular dynamics simulations of WT and G340S *PfFNR*

To explore the intrinsic dynamic profile of WT and G340S *PfFNR*, protein-only molecular dynamics (MD) simulations were performed in GROMACS 2026.0 using a scripted and reproducible workflow ( Abraham et al., 2015; 2026). The WT and G340S starting structures were prepared separately. Topologies were generated with the molecular dynamics simulation suite (gmx) pdb2gmx using the AMBER14SB force field and the TIP3P water model, with hydrogens regenerated during preprocessing. Each protein was centered in a dodecahedral simulation box with a minimum solute-to-box distance of 1.0 nm using gmx editconf, solvated with gmx solvate using the spc216 solvent configuration, neutralized, and adjusted to 0.15 M NaCl using gmx genion. To avoid duplication during preprocessing, any pre-existing solvent and ion entries were removed from the topology before solvation. Energy minimization was carried out using the steepest-descent algorithm for up to 50,000 steps, with convergence defined as a maximum force below 1000 kJ mol ^−1^ nm ^−1^. This was followed by 100 ps of NVT equilibration and 100 ps of NPT equilibration, both with a 2 fs time step. Bonds involving hydrogen atoms were constrained with LINCS. Temperature was maintained at 300 K using the velocity-rescale thermostat, with separate coupling groups for protein and non-protein atoms, and pressure was maintained at 1 bar using isotropic C-rescale coupling. Long-range electrostatics were treated using particle mesh Ewald (PME), with the Verlet cutoff scheme and a 1.2 nm real-space cutoff for both Coulombic and van der Waals interactions. No positional restraints were applied during the production phase. An exploratory and trajectory-specific MD analysis was performed in a single 50 ns production run for each construct. The simulations were therefore used to compare conformational trends under a common protocol rather than to establish replicate-supported dynamic behaviour. After simulation, the run files were processed to remove periodic boundary effects and center the protein. Backbone RMSD was calculated with gmx rms, residue-wise C-alpha RMSF with gmx rmsf -res, radius of gyration with gmx gyrate, and solvent-accessible surface area (SASA) with gmx sasa. Summary statistics were generated from the resulting XVG files using a companion Python script. For RMSD, radius of gyration, and SASA, mean, standard deviation, minimum, maximum, and sample size were calculated over the final 10 ns of each simulation. C-alpha RMSF summary statistics were calculated across the full residue-wise series. Combined WT and G340S summary values were exported for visualization.

### Molecular docking of NADPH and NADP ^+^ to WT and G340S *PfFNR*

To examine whether the G340S substitution altered predicted nicotinamide cofactor recognition, NADPH and NADP ^+^ were docked to FAD-bound WT and G340S *PfFNR* using AutoDock Vina v1.2.7 ( ccsb-scripps, 2025) in a dedicated Conda environment containing Meeko, Open Babel, Biopython, NumPy, pandas, and RDKit ( Cock et al., 2009; Eberhardt et al., 2021; Harris et al., 2020; O’Boyle et al., 2011; Trott & Olson, 2010). Cofactor docking was performed separately with oxidized NADP ^+^ and reduced NADPH. Docking was performed on static FAD-bound receptor models; *PfFNR*_WT_FAD.pdb and *PfFNR*_G340S_FAD.pdb, with ligand input files derived from NADPH and NADP ^+^ SDF structures. Because *PfFNR* functions in the flavin-bound state, FAD was retained throughout the receptor-preparation workflow to preserve a catalytically relevant structural context. Ligands were protonated at pH 7.4, converted to three-dimensional SDF format with Open Babel, and then converted to PDBQT format. During ligand preparation, NADPH produced non-finite charges under the standard charge-assignment workflow; therefore, both NADPH and NADP ^+^ were docked in neutralized form in the final validated protocol. The resulting Vina scores were interpreted as relative docking estimates for comparison between WT and G340S within a common protocol rather than as direct measures of binding free energy. Because the non-standard linked-residue representation of FAD was not handled directly by the standard receptor-preparation routine, each receptor was separated into protein-only and FAD-only components. Protein-only receptor PDBQT files were generated first, after which the FAD moiety was converted separately and merged back with the corresponding protein receptor to generate the final FAD-retained docking receptors, *PfFNR*_WT_FAD.pdbqt and *PfFNR*_G340S_FAD.pdbqt. Residue identity at position 340 was checked before docking to confirm Gly340 in the WT receptor and Ser340 in the mutant receptor.

The docking search space was defined directly from each FAD-bound receptor rather than transferred from an external reference structure. For each model, the centroid of all FAD atoms and the centroid of residue 340 were calculated from the same template-bound PDB file, and the box center was set at the midpoint between these positions. A cubic box of 30 × 30 × 30 Å was used for all final runs. The WT box center was set to (−92.262, 45.146, −27.841), whereas the G340S box center was set to (−90.702, 47.111, −26.719). Docking used the default Vina scoring function with exhaustiveness 96, 20 output modes, an energy range of 6 kcal mol ^−1^, 12 CPU threads, and a fixed random seed of 340340. The four final cofactor-docking runs were WT-NADPH, WT-NADP ^+^, G340S-NADPH, and G340S-NADP ^+^, and the mode 1 Vina score was retained as the best predicted docking score for each complex.

### Protein-protein docking of WT and G340S *PfFNR* with *PfFd*

To assess whether the G340S substitution altered the preferred *PfFNR-PfFd* interaction interface, protein-protein docking was performed using the HADDOCK 2.4 web server hosted on the Bonvin Lab/WeNMR platform ( Dominguez et al., 2003; Honorato et al., 2024). The ferredoxin binding partner was prepared from *Plasmodium falciparum* ferredoxin ( *PfFd*; PDB ID: 1IUE), whereas WT and G340S *PfFNR* receptor structures were prepared locally before submission. Docking was performed on static FAD-bound receptor models, *PfFNR*_WT_FAD.pdb and *PfFNR*_G340S_FAD.pdb. Protein–protein docking submissions used protein-only files containing ATOM records only. In parallel, holo reference structures preserving the FAD-containing *PfFNR* state and the *PfFd* iron-sulfur center were retained for downstream structural interpretation. Two independent HADDOCK docking jobs were submitted: WT *PfFNR* versus *PfFd* and G340S *PfFNR* versus *PfFd.* The same docking parameters were applied to both runs to ensure direct comparison. Active residues for *PfFNR* were defined as 98, 290, and 308, and passive residues were 72, 96, 99, 100, 102, 167, 168, 255, 256, 257, 258, 285, 286, 288, 289, 292, 293, 294, 304, 306, 307, 309, and 310. Active residues for PfFd were 26, 29, 34, 65, and 66, and passive residues were 9, 10, 22, 23, 30, 31, 32, 33, 35, 36, 37, 40, 61, 62, 63, 64, 67, 69, 70, 71, 89, 90, and 98. Additional parameters included random seed 917, ntrials = 5, anastruc_1 = 200, 500 rigid-body docking models, 1000 semi-flexible refinement models, and 1000 water-refined models. FCC-based clustering was applied with clust_cutoff = 0.6 and clust_size = 4, and automatic passive residue generation was enabled for both docking partners. Post-docking analysis was based on cluster ranking, cluster membership, RMSD distributions, buried surface area, and restraint-violation summaries extracted from the HADDOCK output tree.

### Molecular docking of antimalarial ligands to WT and G340S *PfFNR*

To examine whether the G340S substitution altered broader ligand accommodation within the cofactor-associated region, four antimalarial compounds-lumefantrine (LM) CHEBI: 156095, primaquine (PMQ) CHEBI: 8405, artemisinin (ART) CHEBI: 223316, and dihydroartemisinin (DHA) CHEBI: 207229 were docked to the same FAD-bound WT and G340S *PfFNR* receptor models used for cofactor docking. Docking was performed in the same computational environment and with the same AutoDock Vina v1.2.7 workflow described above ( Eberhardt et al., 2021; Trott & Olson, 2010). FAD was retained in both receptor models to evaluate ligand placement in the context of the cofactor-bound enzyme state. Ligands were prepared from SDF input files before conversion to PDBQT format. LUM and PMQ were protonated at pH 7.4 and converted to three-dimensional conformers before ligand preparation. For ART and DHA, fresh three-dimensional conformers were generated and energy minimized with the MMFF94 force field before PDBQT conversion because the initial ART input produced an invalid three-dimensional representation incompatible with Vina. The receptor-specific search spaces defined for cofactor docking were retained unchanged for all four antimalarial ligands so that all comparisons were made within a common docking protocol. Thus, WT docking used a box centred at (−92.262, 45.146, −27.841), and G340S docking used a box centred at (−90.702, 47.111, −26.719), with a box size of 30 × 30 × 30 Å in both cases. Each docking run used exhaustiveness 96, 20 output modes, an energy range of 6 kcal mol ^−1^, 12 CPU threads, and a random seed of 340340. Eight receptor-ligand combinations were therefore evaluated: WT-LM, WT-PMQ, WT-ART, WT-DHA, G340S-LM, G340S-PMQ, G340S-ART, and G340S-DHA. For each complex, the mode-1 Vina score was retained as the best-docking score, and the corresponding pose was used for downstream structural inspection in ChimeraX ( Meng et al., 2023).

### Data repository

All the dataset utilized and generated in this manuscript have been deposited in https://zenodo.org/uploads/17010754. DOI: 10.5281/zenodo.17010754 ( Kiboi & Muriithi, 2025). An extra extended dataset utilised and generated during additional analysis for NADPH and NADP ^**+**^ docking, MD simulation, ligand (drug) docking and *PfFNR-PfFd* binding using haddock has been deposited in https://zenodo.org/records/20217529. DOI: 10.5281/zenodo.20217529 ( Kiboi & Muriithi, 2026).

## Results

### Conservation of G340 of *P. falciparum* FNR

**Figure 2.**
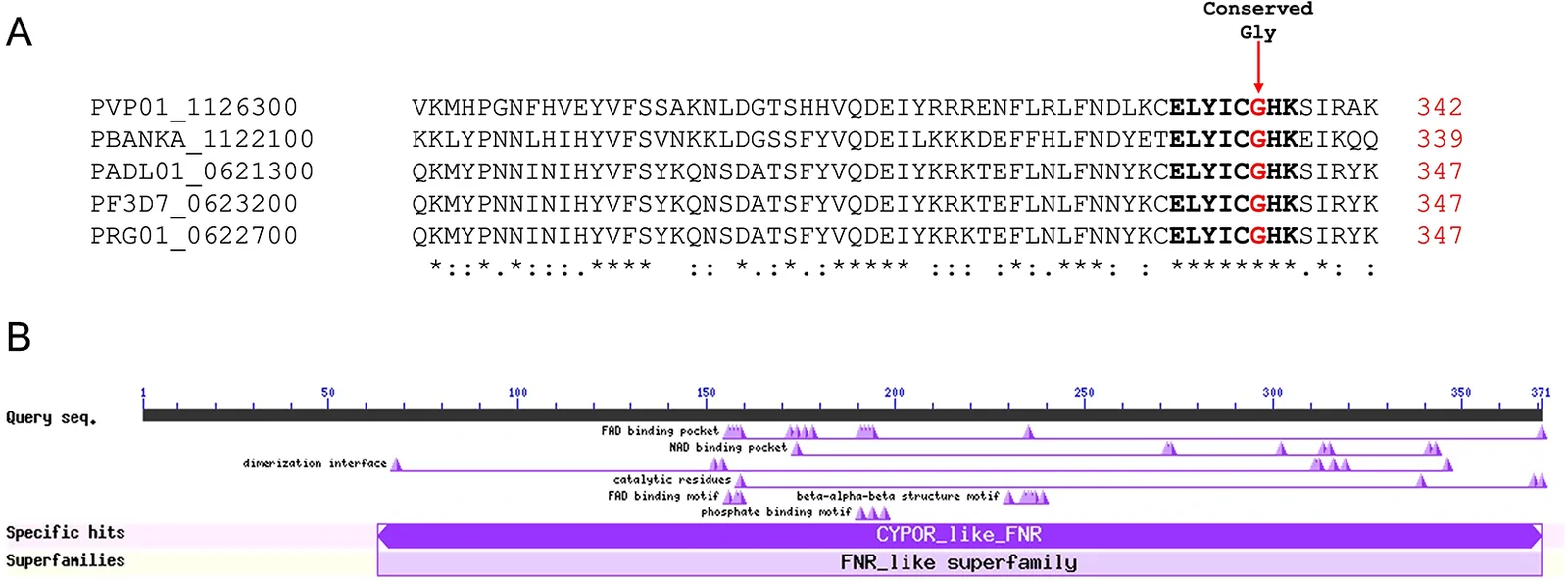
Conservation and domain context of G340 in *Plasmodium falciparum* ferredoxin NADP ^+^ reductase. **(A)** Multiple sequence alignment of FNR orthologs from representative Plasmodium species showing conservation of the glycine residue (red) corresponding to G340 in *PfFNR*. **(B)** Domain and motif context of *PfFNR* showing the position of G340 relative to the FNR-like/CYPOR-like region, the FAD/NADP ^+^-associated catalytic region, and the ferredoxin-interaction region.

**Figure 3.**
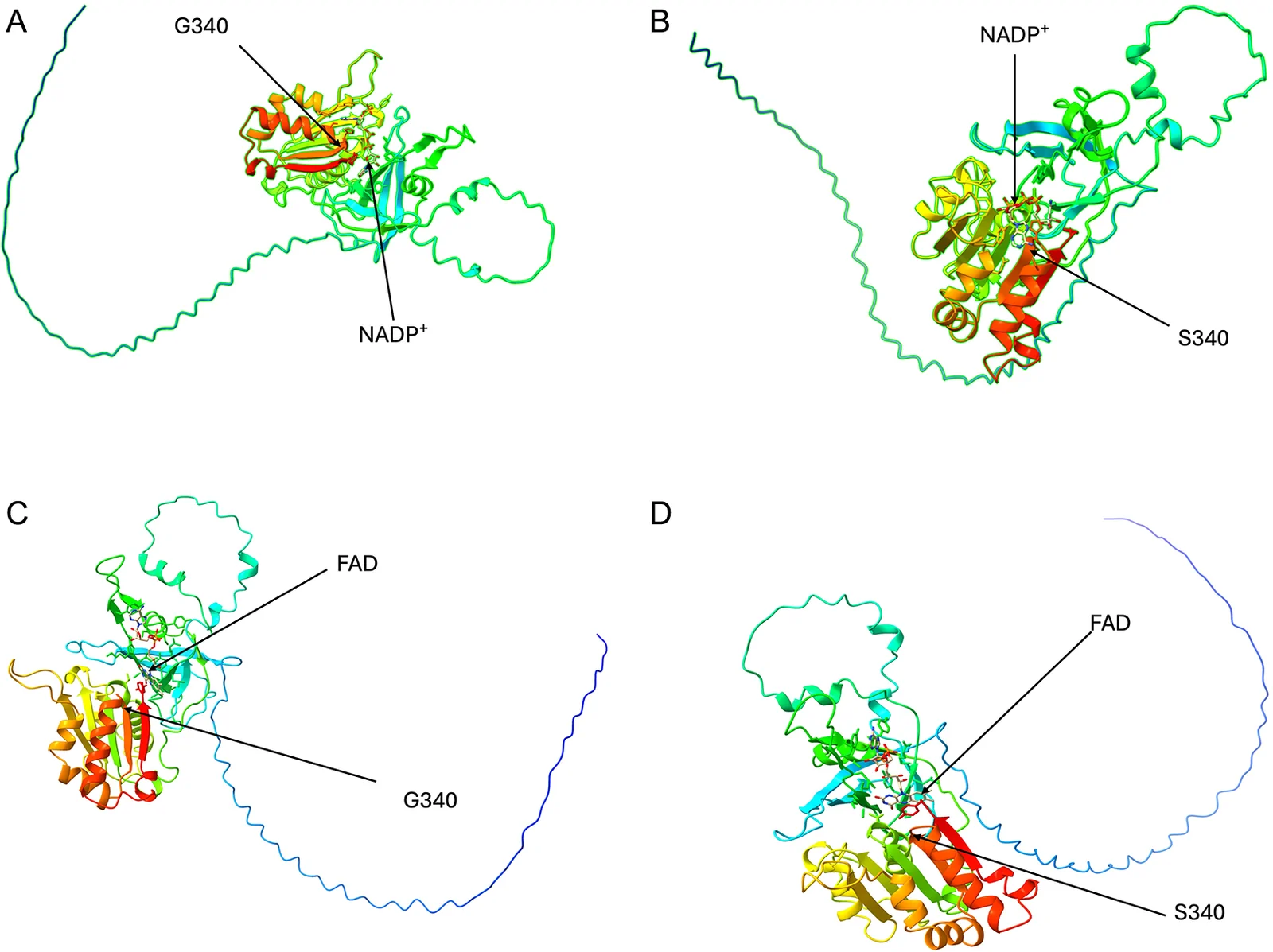
Structural mapping of residue 340 in WT and G340S *PfFNR* models. AlphaFold 3-derived structural models showing the location of residue 340 relative to the nicotinamide cofactor-binding region and the FAD-associated catalytic environment. **(A)** WT *PfFNR* model showing G340 near the NADP ^+^-binding region. **(B)** G340S *PfFNR* model showing S340 in the corresponding NADP ^+^-binding region. **(C)** WT *PfFNR* model showing G340 in relation to the FAD-associated catalytic environment. **(D)** G340S *PfFNR* model showing S340 in relation to the FAD-associated catalytic environment.

Multiple sequence alignment of *FNR* orthologs from *P. falciparum*, *P. berghei*, *P. vivax*, *P. reichenowi*, and *P. adleri* showed strong conservation across the catalytic FAD/NADP ^+^ reductase region, with more than 85% sequence identity within the core domain and preservation of residues involved in flavin and nicotinamide cofactor recognition ( Figure 2A). Gly340 in *PfFNR* was invariant across all sequences examined. Conserved-domain and motif analyses further placed G340 within the FNR-like/CYPOR-like region on a loop adjacent to the NADP ^+^ binding site, together with canonical plant-type FNR FAD/ NADP ^+^ binding domains and the ferredoxin-interaction region ( Figure 2B). Although the canonical GxGxxG motif was not retained in strict sequence form, motif- and domain-based analyses supported preservation of the Rossmann-type oxidoreductase architecture. Together, these findings place G340 in a structurally constrained region of *PfFNR* and support further analysis of the G340S substitution. An initial probe of the structural context of the G340S substitution using AlphaFold3 yielded high-confidence three-dimensional models of WT and G340S *PfFNR* ( Figure 3). Both models showed mean per-residue pLDDT scores of approximately 90–92, indicating high local structural reliability, particularly across the FAD/NADP ^+^ binding domain. Consistent with this high-confidence prediction, both WT and G340S *PfFNR* retained a closely comparable overall fold, with no evidence of gross structural disruption following substitution of Gly340 with serine ( Figure 3A, B). Mapping of residue 340 onto the predicted structures further showed that both Gly340 in the WT model and Ser340 in the mutant model were positioned in close spatial proximity to the NADP ^+^ binding region and adjacent to the FAD-containing catalytic environment ( Figure 3C, D).

### Structural superimposition shows preservation of the global *PfFNR* fold with localized changes around residue 340

**Figure 4.**
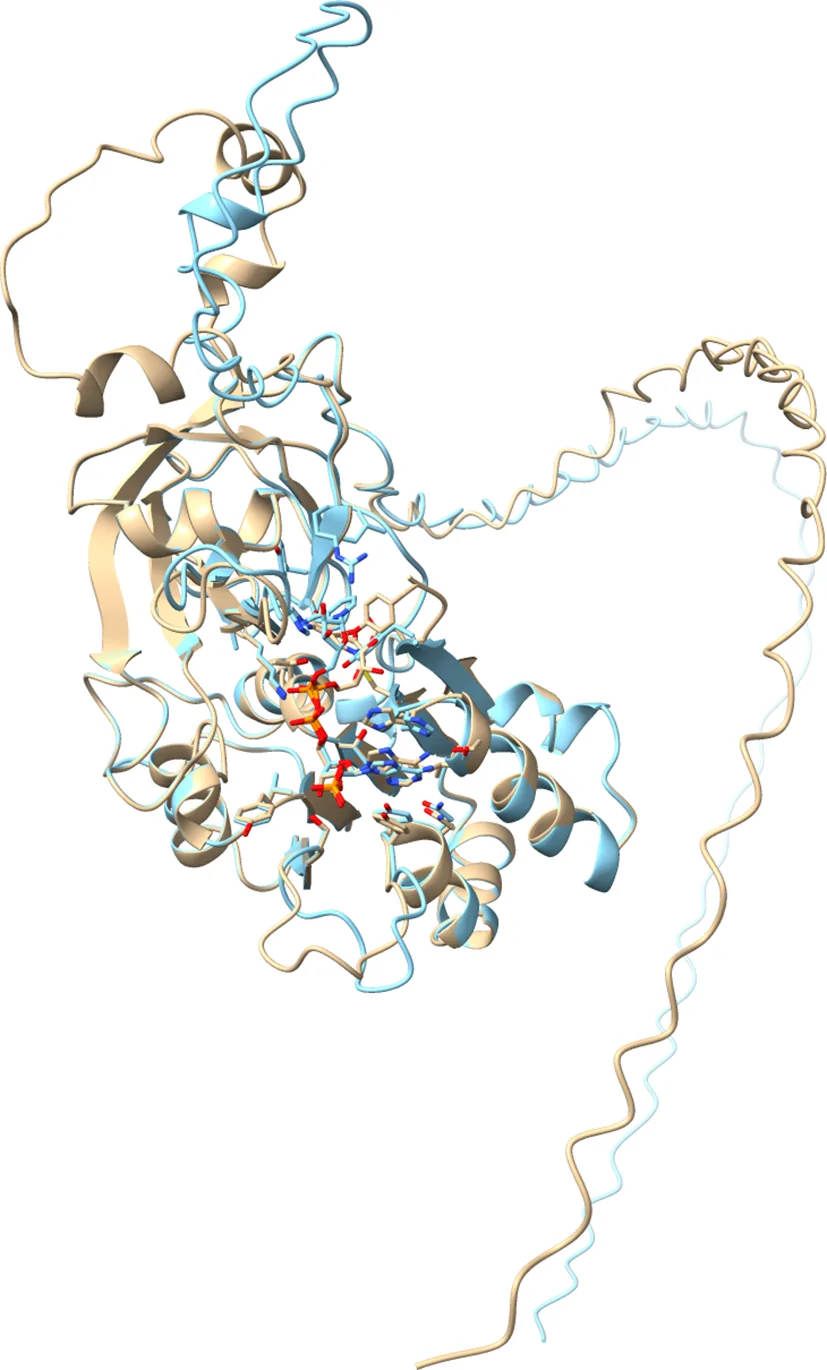
Structural superposition of WT and G340S *PfFNR* models.

Structural superimposition of the WT and G340S *PfFNR* models showed that the G340S substitution did not cause major disruption of the overall enzyme fold. UCSF Chimera alignment of the shared structured region returned an RMSD of 0.758 Å across 331 aligned Cα atom pairs. After iterative outlier rejection through pruning, 325 Cα atom pairs were retained, and the refined RMSD decreased to 0.649 Å ( Figure 4; Table 1). These low RMSD values indicate strong structural similarity between the WT and mutant models, particularly within the conserved *PfFNR* core. The structural overlay showed close agreement across the central catalytic and cofactor-associated region, whereas the largest visible deviations were restricted mainly to peripheral loops and extended surface segments. A focused view of the mutation site showed only subtle positional differences around residue 340 and the adjacent cofactor-associated environment. The Q-score of 0.748 further supported substantial global structural correspondence between the two models, while the SDM value of 15.781 indicated measurable but localized structural divergence rather than widespread structural perturbation. Together, these analyses indicate that G340S preserves the overall *PfFNR* scaffold while introducing only limited local conformational changes near residue 340 and in the cofactor-binding region.

**Table 1.** Structural evaluation metrics from superimposition of wild-type (WT) and G340S *PfFNR* models.

Category	Metric	Description	PfFNR WT vs PfFNR G340S (AlphaFold3)
**Structural Metrics**	RMSD (Å)	Backbone deviations between WT and mutant models	0.758
	Pruned RMSD (Å)	RMSD after outlier residue removal	0.649
	Q-score	Global structural similarity index	0.748
	SDM score	Local structural deviations (mutation impact)	15.781

**RMSD (Å)**: Root mean square deviation; lower values indicate closer structural similarity. **Pruned RMSD (Å)**: RMSD calculated after removing flexible or outlier residues. **Q-score **: Global structural similarity index (0–1 scale, higher = more similar). **SDM score**: Structural deviation metric; higher values indicate greater perturbation due to mutation.

### Exploratory single-run molecular dynamics analysis suggests that G340S may alter *PfFNR* conformational sampling

**Figure 5.**
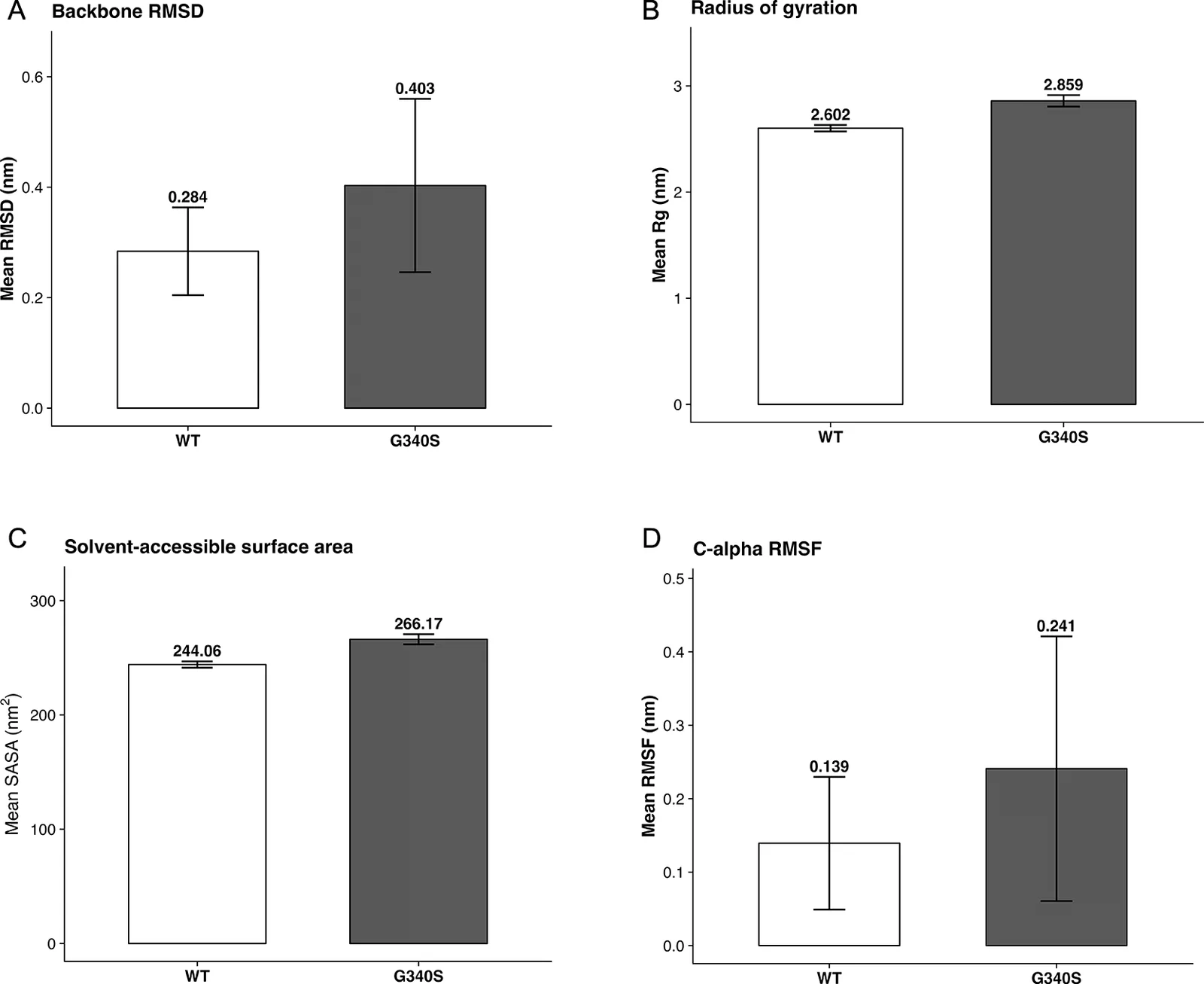
Molecular dynamics summary of WT and G340S *PfFNR.* Comparison of structural dynamics between WT and G340S *PfFNR* from 50 ns protein-only molecular dynamics simulations. **(A)** Mean backbone RMSD over the final 10 ns of simulation. **(B)** Mean radius of gyration (Rg), reflecting global compactness. **(C)** Mean solvent-accessible surface area (SASA), reflecting solvent exposure. **(D)** Mean C-alpha RMSF, reflecting residue-level flexibility.

To explore whether the localized structural differences observed in the mutant could be associated with altered conformational behaviour, single 50 ns molecular dynamics runs of WT and G340S PfFNR were compared using backbone RMSD, radius of gyration (Rg), solvent-accessible surface area (SASA), and residue-level C-alpha RMSF ( Figure 5A-D; Table S1). Across all four descriptors, the G340S mutant showed higher values than WT, consistent with greater structural deviation, reduced compactness, increased solvent exposure, and enhanced local flexibility under the present simulation conditions. Backbone RMSD over the final 10 ns was higher in G340S than in WT (0.40301 ± 0.15696 nm versus 0.28388 ± 0.07950 nm), representing an increase of approximately 42%. The mutant also reached a higher maximum RMSD (0.70398 nm) than WT (0.40970 nm), indicating greater deviation from the starting structure ( Figure 5A). Global compactness followed the same pattern: the mean Rg was higher in G340S than in WT (2.85914 ± 0.05376 nm versus 2.60158 ± 0.03035 nm), corresponding to an increase of approximately 9.9% ( Figure 5B). SASA was likewise elevated in the mutant (266.16745 ± 4.40316 nm ^2^ versus 244.05646 ± 2.74483 nm ^2^), corresponding to an increase of approximately 9.1% and indicating greater solvent exposure ( Figure 5C). Residue-level flexibility was also increased in G340S, with a higher mean C-alpha RMSF than WT (0.24088 ± 0.18018 nm versus 0.13939 ± 0.09034 nm), corresponding to an increase of approximately 72.8%, together with a markedly higher maximum RMSF (1.11040 nm versus 0.63820 nm) ( Figure 5D). Under the present single-run simulation conditions, the G340S substitution was associated with trends toward a more dynamic and conformationally expanded *PfFNR* state; however, replicate simulations will be required to determine whether these differences are reproducible.

### G340S selectively alters predicted nicotinamide cofactor recognition

**Figure 6.**
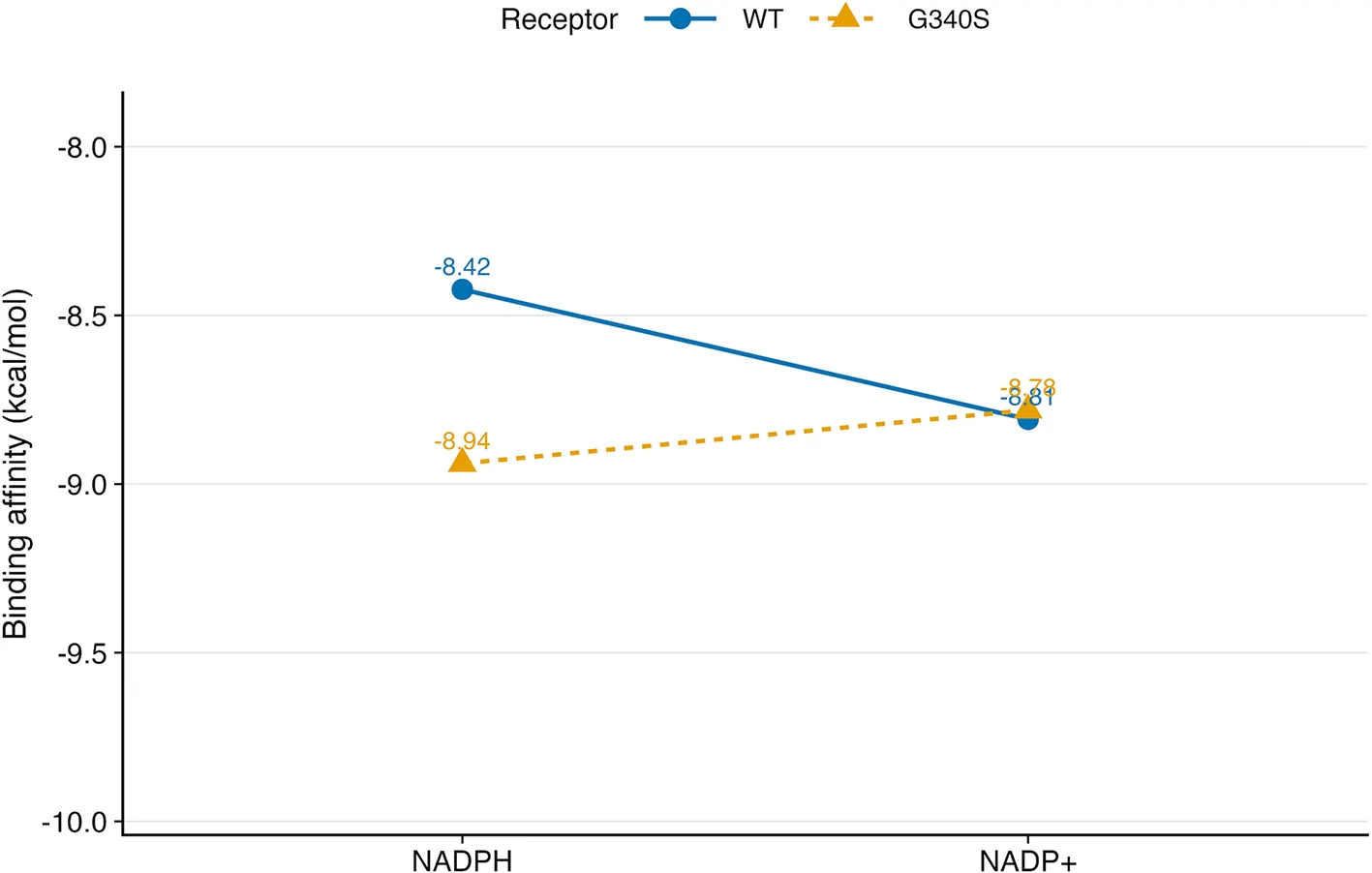
Predicted docking scores of NADPH and NADP ^+^ to WT and G340S *PfFNR*. More negative values indicate more favorable predicted docking scores. Lines are included only to aid visual comparison between receptor states and should not be interpreted as a continuous trend.

Docking of NADPH and NADP ^+^ to the FAD-bound WT and G340S *PfFNR* models yielded favorable predicted binding energies for all four complexes ( Figure 6; Table S2). In the WT receptor, NADP ^+^ docked slightly more favorably than NADPH, with best predicted affinities of −8.808 and − 8.423 kcal/mol, respectively. In the G340S receptor, NADPH docked more favorably than in WT, with a best predicted affinity of −8.939 kcal/mol, whereas NADP ^+^ docking was essentially unchanged at −8.781 kcal/mol (Table S2). Comparison of the two receptor states showed that the mutation had a selective effect on cofactor docking. In WT *PfFNR*, NADP ^+^ was favored over NADPH by 0.385 kcal/mol, whereas in the mutant, NADPH was slightly favored over NADP ^+^ by 0.158 kcal/mol. The clearest mutation-associated shift was therefore observed for NADPH, while predicted NADP ^+^ recognition remained largely stable. These results suggest that G340S does not abolish nicotinamide cofactor binding but instead induces a modest shift in predicted cofactor preference, with the stronger effect seen for NADPH ( Figure 6; Table S2).

### HADDOCK analysis supports altered *PfFNR–PfFd* docking preferences in G340S *PfFNR*

**Figure 7.**
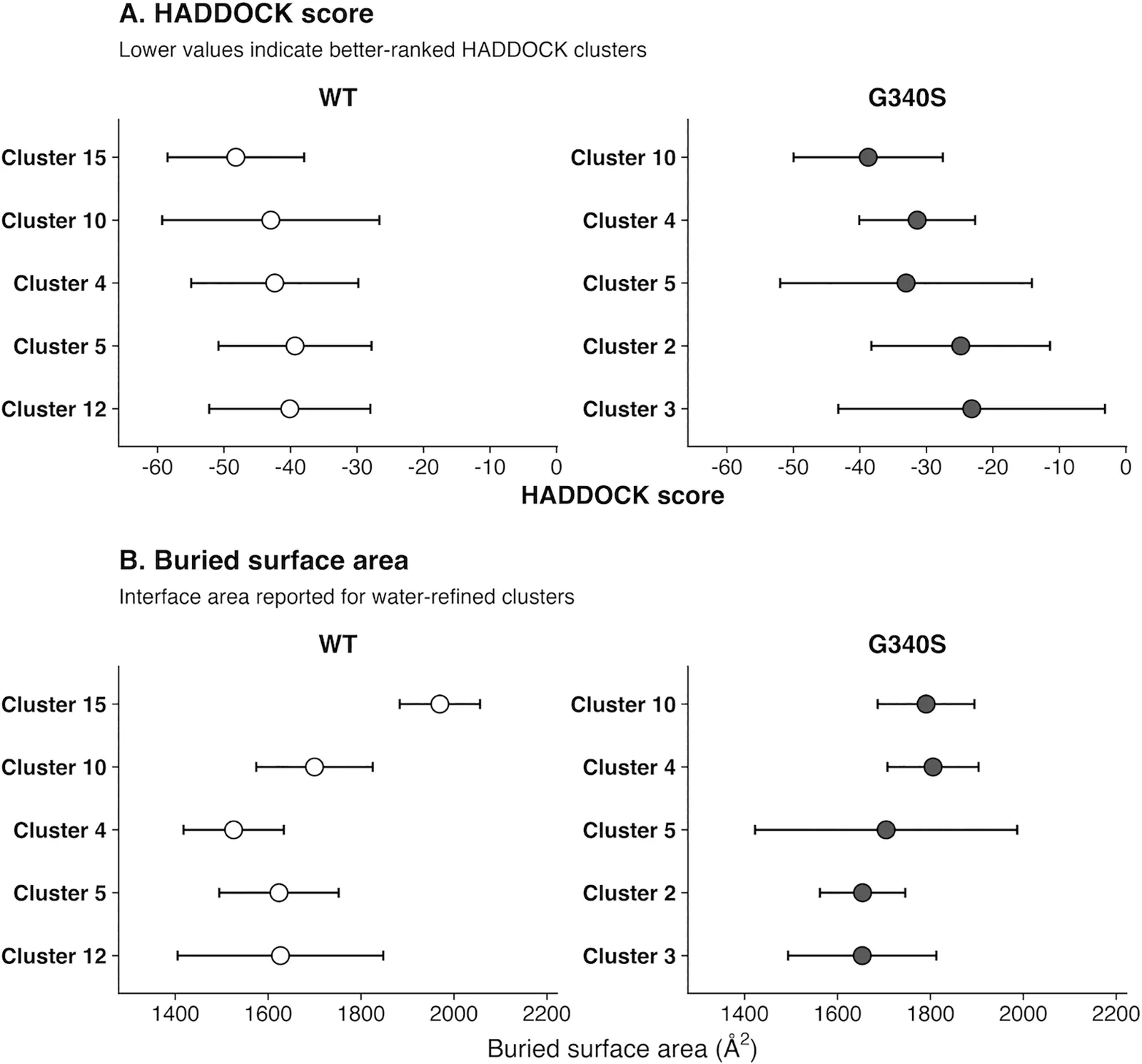
HADDOCK-derived docking score and buried interface comparisons for WT and G340S *PfFNR*– *PfFd* complexes. **(A)** HADDOCK scores for selected refined docking clusters from WT and G340S *PfFNR* in complex with *PfFd*. More negative HADDOCK scores indicate more favorable predicted docking solutions. **(B)** Buried surface area values for the corresponding refined clusters, reported in Å ^2^.

To compare predicted *PfFd* docking against WT and G340S *PfFNR*, water-refined HADDOCK clustering was performed for each receptor state ( Figure 7; Table S3). Clusters were ranked within each system according to HADDOCK score, with lower values representing better-ranked docking solutions. In the WT docking run, cluster 15 was the leading solution, with a HADDOCK score of −48.223 ± 10.274 and a buried surface area of 1969.527 ± 86.477 Å ^2^. The remaining WT clusters showed progressively less favourable HADDOCK scores: cluster 10 scored −42.968 ± 16.332, cluster 4 scored −42.379 ± 12.568, cluster 12 scored −40.120 ± 12.117, and cluster 5 scored −39.330 ± 11.511. The buried surface areas for these WT clusters ranged from 1525.729 ± 107.886 Å ^2^ for cluster 4 to 1699.765 ± 125.391 Å ^2^ for cluster 10, indicating that WT *PfFNR* sampled multiple *PfFd* docking arrangements with variable interface sizes. The G340S mutant displayed a different distribution of HADDOCK-ranked clusters. Cluster 10 was the best-ranked G340S solution, with a HADDOCK score of −38.758 ± 11.224 and a buried surface area of 1790.722 ± 104.088 Å ^2^. Other G340S clusters were less favourably ranked, with cluster 5 scoring −33.063 ± 18.930, cluster 4 scoring −31.390 ± 8.711, cluster 2 scoring −24.841 ± 13.436, and cluster 3 scoring −23.190 ± 20.045. Buried surface areas in the G340S run ranged from 1653.201 ± 159.460 Å ^2^ for cluster 3 to 1805.700 ± 98.054 Å ^2^ for cluster 4. Although G340S cluster 4 showed a slightly larger buried surface area than cluster 10, its HADDOCK score was less favourable, indicating that interface size alone did not determine the top-ranked docking solution. Overall, direct comparison of the leading clusters showed that the best-ranked WT solution had both a more favourable HADDOCK score and a larger buried interface than the best-ranked G340S solution.

### G340S differentially affects docking of antimalarial ligands to *PfFNR*

**Figure 8.**
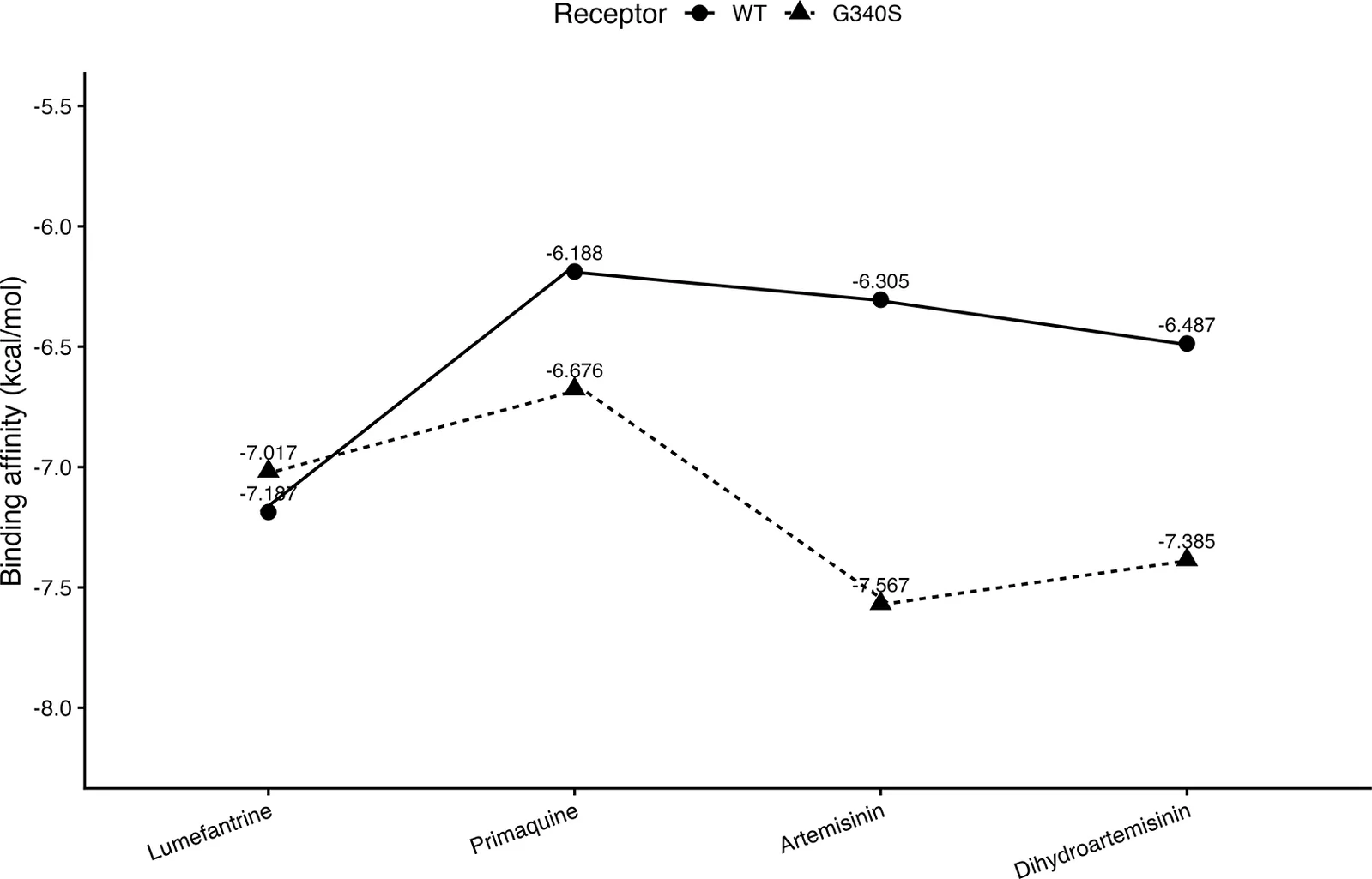
Comparative docking of selected antimalarial ligands to WT and G340S *PfFNR*. Values are reported as predicted docking scores in kcal/mol, with more negative values indicating more favorable predicted docking. Lines are included only to aid visual comparison across ligands and receptor states.

**Figure 9.**
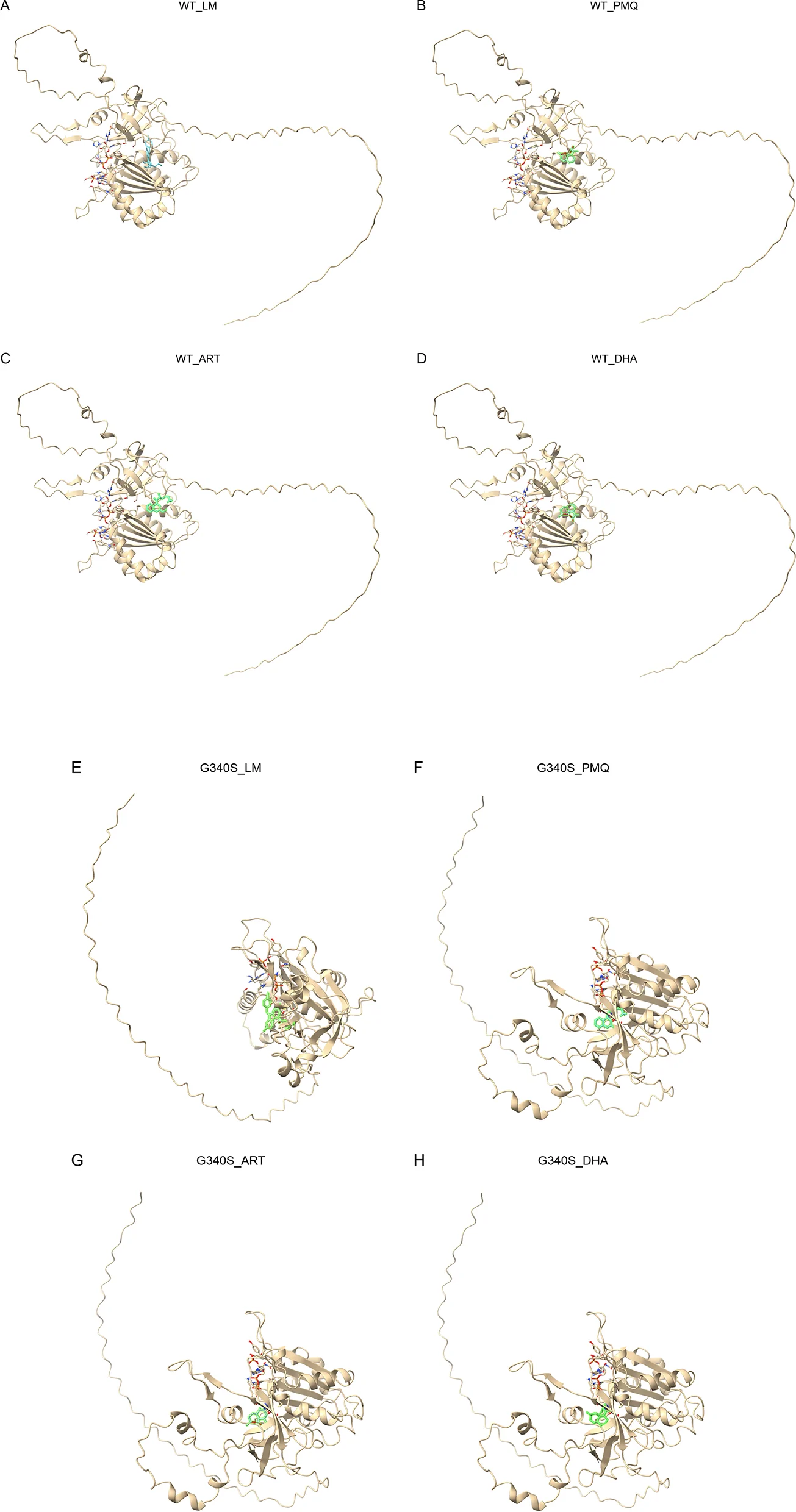
Representative docking poses of antimalarial ligands within the WT and G340S *PfFNR*. Mode-1 docking poses of four antimalarial compounds docked to *Plasmodium falciparum* ferredoxin NADP ^+^ reductase ( *PfFNR*). **(A–D)** Representative poses in the WT *PfFNR* receptor: (A) lumefantrine, (B) primaquine, (C) artemisinin, and (D) dihydroartemisinin. ( **E–H)** Representative poses in the G340S *PfFNR* receptor: (E) lumefantrine, (F) primaquine, **(G)** artemisinin, and **(H)** dihydroartemisinin.

All four antimalarial ligands docked within the predefined FAD-proximal region of both WT and G340S *PfFNR*, but predicted docking scores varied by ligand identity and receptor background ( Figure 8). In the WT receptor, lumefantrine showed the most favourable mode-1 docking score, −7.084 kcal mol ^−1^, followed by dihydroartemisinin, −6.487 kcal mol ^−1^, artemisinin, −6.305 kcal mol ^−1^, and primaquine, −6.144 kcal mol ^−1^. In the G340S receptor, the ranking shifted, with artemisinin showing the most favourable predicted score, −7.567 kcal mol ^−1^, followed by dihydroartemisinin, −7.385 kcal mol ^−1^, lumefantrine, −7.017 kcal mol ^−1^, and primaquine, −6.676 kcal mol ^−1^. The effect of G340S on predicted ligand docking was ligand-dependent. Lumefantrine was largely unchanged between WT and G340S, differing by only 0.067 kcal mol ^−1^, whereas primaquine showed a modest shift towards a more favourable predicted docking score in the mutant receptor, changing from −6.144 to −6.676 kcal mol ^−1^. Notably, the largest differences were observed for the endoperoxide compounds. Artemisinin shifted from −6.305 kcal mol ^−1^ in WT to −7.567 kcal mol ^−1^ in G340S, while dihydroartemisinin shifted from −6.487 to −7.385 kcal mol ^−1^. Inspection of the retained mode-1 poses showed that the WT ligands occupied the FAD-adjacent docking region with ligand-specific orientations ( Figure 9A–D), whereas the corresponding G340S complexes showed altered placement within the same predefined search region ( Figure 9E–H).

## Discussion

The results from this study provide general predictive *in silico* insights on the impact of the G340S substitution in *PfFNR.* The results show that G340S is unlikely to be a neutral substitution in *PfFNR.* The mutation preserves the overall fold of the enzyme, yet the combined structural, dynamics, cofactor-docking, protein-protein docking, and ligand-docking results point to a more specific mechanism involving local remodelling near a functionally important cofactor-associated region, altered conformational behaviour, a shift in predicted nicotinamide cofactor accommodation, changes in the predicted *PfFNR–PfFd* docking profile, and ligand-dependent effects within the FAD-proximal pocket. Interpreting these findings requires viewing Gly340 in the context of *PfFNR* function rather than as an isolated conserved residue. Our focus on the Gly340 position was informed by earlier lumefantrine-selection studies in *P. berghei*, in which an analogous FNR substitution emerged under drug pressure ( Kiboi et al., 2020). Although rodent malaria models do not reproduce all aspects of *P. falciparum* infection, they remain useful for identifying conserved parasite pathways that respond to antimalarial selection and for generating mechanistic hypotheses that can then be tested in the human parasite ( Borges et al., 2011; Hunt et al., 2007; Kiboi et al., 2014; Kiboi et al., 2009). In that sense, the value of the *P. berghei* work is not to prove an identical resistance mechanism in *P. falciparum*, but to identify the homologous *PfFNR* G340 position as a biologically credible site for further analysis in the context of lumefantrine response. This interpretation is strengthened by the conservation data, which place Gly340 within a region of *PfFNR* likely to be functionally constrained. Therefore, the conservation analysis places Gly340 in a biologically meaningful context. *PfFNR* sits at the core of the apicoplast ferredoxin/FNR redox pathway, transferring reducing equivalents from NADPH to ferredoxin to support essential plastid metabolism ( Akuh et al., 2022; Balconi et al., 2009). Biochemical and structural studies have shown that the *PfFNR* NADP(H) binding region differs in important ways from canonical plant-type FNRs, and that modest perturbations in this region can alter cofactor recognition and catalysis ( Baroni et al., 2012; Crobu et al., 2009). In this context, the complete conservation of Gly340 across the examined *Plasmodium* FNR orthologs is most consistent with structural or functional constraint rather than evolutionary neutrality. The most likely role of this G340 residue would therefore be to help preserve the local conformation and flexibility of a sensitive loop environment near the nicotinamide cofactor-binding surface. If that is the case, the next question is whether replacing glycine with serine produces a detectable structural effect while leaving the overall *PfFNR* scaffold intact. Structural superimposition addresses this point by showing that G340S is not best interpreted as a globally disruptive mutation. Instead, the comparison supports a localized structural effect near residue 340, with potential consequences for cofactor positioning, local packing, and interaction-surface geometry. Chemically, this interpretation is plausible because glycine can support conformational flexibility, whereas serine introduces a polar side chain that may alter hydrogen bonding, packing, or loop behaviour ( Lopez & Mohiuddin, 2024). The superimposition findings therefore provide a structural bridge between Gly340 conservation and the downstream analyses, suggesting that G340S may influence *PfFNR* function by reshaping a local functional environment while preserving the overall enzyme scaffold. However, because superimposition is a static comparison, it cannot determine whether this perturbation affects conformational behaviour over time. The molecular dynamics analyses, therefore, provide the next level of evidence by testing whether the mutant samples prefer a different structural state under the present simulation conditions.

The molecular dynamics results further extend understanding of whether the mutant samples prefer a different structural state under simulation conditions, suggesting that G340S shifts *PfFNR* toward a broader conformational ensemble rather than simply destabilising the protein. The combined increases in RMSD, radius of gyration, SASA, and RMSF are consistent with a mutant that is more flexible, less compact, and more solvent-exposed under the present simulation conditions. For a redox enzyme such as *PfFNR*, productive catalysis depends on coordinated positioning of the FAD-associated core, the nicotinamide cofactor-binding region, and the ferredoxin-facing surface ( Swift et al., 2022). Even modest changes in conformational sampling could therefore shift the balance between productive and nonproductive states. The present data support a shift in the dynamic landscape of *PfFNR* rather than a simple stable-versus-unstable model. That distinction is important because changes in conformational amino acid residues would be expected to influence not only the global *PfFNR* profile, but also how the cofactor-binding region accommodates NADPH and NADP ^+^. The cofactor-docking results fit the same general interpretation. G340S did not abolish predicted recognition of either NADPH or NADP ^+^, arguing against a substantial loss of cofactor competence. Instead, the stronger effect was seen for NADPH, suggesting that the mutation subtly changes how the cofactor-binding region accommodates the reduced nicotinamide species. This is a biologically relevant outcome for a residue positioned near this functional surface, and it agrees with earlier work showing that relatively small perturbations in the *PfFNR* NADP(H) binding region can affect cofactor interaction and catalytic activity ( Baroni et al., 2012; Crobu et al., 2009). It is also broadly consistent with reports that dihydroartemisinin partially inhibits *PfFNR* diaphorase activity by reducing affinity for NADPH while leaving electron transfer to ferredoxin largely intact, indicating that this redox system is susceptible to selective perturbation ( Kimata-Ariga et al., 2019; Kimata-Ariga & Morihisa, 2022). A shift in the cofactor-accommodation is not, however, definitive evidence that G340S changes catalytic preference.

In the *PfFNR-PfFd* redox system, cofactor handling and partner recognition are functionally coupled; it is reasonable to ask whether G340S substitution also reshapes the predicted interaction landscape with *PfFd.* The *PfFNR–PfFd* docking analysis suggests that the consequences of G340S may extend beyond cofactor-associated structural changes to altered partner-protein docking behaviour. The key biological question is not simply whether *PfFd* can associate with *PfFNR*, but whether the two proteins adopt orientations compatible with efficient electron transfer. In the present HADDOCK analysis, the best-ranked WT cluster showed both a more favourable HADDOCK score and a larger buried interface than the best-ranked G340S cluster, while both systems retained multiple ranked docking solutions. This pattern supports a change in the predicted docking profile rather than a simple gain in *PfFd* binding by the mutant. HADDOCK score is a composite term that incorporates intermolecular van der Waals, electrostatic, desolvation, and restraint-energy contributions ( Honorato et al., 2024). Water-refined clusters are ranked using the average HADDOCK score of selected models within each cluster, whereas buried surface area is reported as an interface descriptor rather than the sole determinant of cluster ranking ( Honorato et al., 2024; van Zundert et al., 2016). The observation that some clusters have relatively large, buried interfaces but less favourable HADDOCK scores is therefore not unexpected and should be interpreted in the context of the full scoring function. The biological relevance of these docking differences is supported by the role of *PfFNR* and *PfFd* as an apicoplast redox pair. *PfFNR* catalyzes NADPH-dependent reduction of *PfFd*, which then distributes reducing equivalents to different apicoplast-associated pathways, including iron–sulfur protein-dependent metabolic reactions and other essential biosynthetic processes ( Akuh et al., 2022; Balconi et al., 2009; Lesanavičius et al., 2020; Milani et al., 2007). Previous biochemical and NMR-based work has shown that *PfFd–PfFNR* recognition depends on defined acidic regions on *PfFd*, including Asp26, Glu29, and Glu34, with Glu29 reported as particularly important for interaction with *PfFNR* ( Kimata-Ariga et al., 2007). Thus, the current HADDOCK results do not support stronger *PfFd* docking by G340S. Instead, they indicate that the substitution is associated with a redistribution of predicted *PfFNR–PfFd* docking solutions, with the mutant retaining multiple accessible docking arrangements but showing a less favourable top-ranked HADDOCK score and a smaller buried interface than WT. A residue that affects both cofactor accommodation and predicted partner docking might also be expected to influence how the FAD-proximal region accommodates small molecules.

The expectation that cofactor accommodation and predicted partner docking impact binding of small molecules is broadly consistent with the ligand (drug)-docking results. The stronger shifts observed for artemisinin and dihydroartemisinin, together with a more modest shift for primaquine and only minimal change for lumefantrine, suggest that G340S does not simply create a more permissive cavity; rather, it appears to alter local binding-space features in a chemotype-dependent manner. This is particularly interesting considering previous work shows that DHA can affect the *PfFNR/Fd* redox system and may interact with the NADPH-associated binding cavity, and that *PfFNR* can participate in redox cycling of other antimalarial chemotypes ( Cichocki et al., 2021; Kimata-Ariga et al., 2019; Kimata-Ariga & Morihisa, 2022; Lesanavičius et al., 2020). Similarly, to exert antimalarial action, hydroxylated primaquine metabolites are activated by FNR ( Vásquez-Vivar & Augusto, 1992). Lumefantrine action, by contrast, is more commonly associated with digestive-vacuole and heme-associated parasite killing than with a defined *PfFNR* interaction ( Vanaerschot et al., 2017). Any contribution of *PfFNR* variation to lumefantrine-response phenotypes is therefore more likely to be indirect, through altered redox adaptation, stress handling, or compensatory metabolic effects, rather than through direct target engagement. This interpretation fits both the minimal docking shift seen for lumefantrine and the broader uncertainty that still surrounds the molecular basis of reduced lumefantrine susceptibility in *P. falciparum* ( Halsey & Plucinski, 2023; Vanaerschot et al., 2017). Taken together, these ligand-dependent effects reinforce the broader view that G340S perturbs a redox-sensitive structural environment rather than a single isolated interaction.

In this study, the modelling strategy was an important foundation because the biological question was mutation-specific. To date, the available structural and biochemical studies of *PfFNR* provide an essential foundation for understanding the WT enzyme, but they do not capture the G340S variant, its altered conformational ensemble, or its potential effects on cofactor recognition, *PfFd* interaction, and ligand accommodation within a single framework. In this setting, AlphaFold3-based structural modelling, molecular dynamics simulations, and docking offered a practical first-pass way to test whether a single residue substitution could plausibly reshape the local architecture and interaction landscape of *PfFNR.* These approaches do not replace recombinant biochemistry or parasite genetics, but they are suited to narrowing the mechanistic possibilities and identifying the most testable hypotheses for follow-up. The interpretation of the present findings should be considered in light of important limitations. This study is a computational investigation and therefore cannot, on its own, establish that G340S changes PfFNR catalysis, parasite fitness, redox homeostasis, or drug susceptibility in vivo. In particular, the molecular dynamics component should be regarded as exploratory because only a single 50 ns production simulation was completed for each construct. Replicate MD simulations would require substantially greater computational resources than were available for the present study. Single MD trajectories can be influenced by stochastic sampling, initial velocity assignment, and incomplete exploration of conformational space; therefore, the observed differences in RMSD, radius of gyration, SASA, and RMSF should be interpreted as trajectory-specific trends under the present simulation conditions rather than as replicate-supported evidence of a reproducible dynamic phenotype. Future studies should include independent replicate simulations with different starting velocity seeds, longer production times, and convergence or ensemble-based analyses to determine whether the observed conformational differences are robust. Similarly, the docking analyses provide structural hypotheses rather than direct measurements of binding affinity or catalytic activity. Biochemical assays, parasite-genetic validation, and drug-susceptibility testing, for example using CRISPR/Cas-based introduction of the G340S variant, will be required to determine whether this substitution has functional relevance. Despite these limitations, the convergence of conservation analysis, structural superimposition, exploratory MD, cofactor docking, PfFNR–PfFd docking, and ligand-docking results supports Gly340 as a biologically credible residue for future functional testing. G340S should therefore be viewed not as a validated antimalarial-response marker, but as a redox-associated candidate variant that warrants direct experimental evaluation.

From a clinical perspective, the importance of this work lies in candidate-marker discovery. If future biochemical and parasite genetic studies confirm that variation at *PfFNR* contributes to altered drug response, then this pathway could become relevant to molecular surveillance of emerging partner-drug resistance in *P. falciparum.* That would be particularly valuable in African settings where artemether-lumefantrine remains widely deployed and especially where validated markers of reduced lumefantrine susceptibility remain limited. More broadly, establishing a redox-associated contributor to altered drug response would expand the current resistance framework beyond the transporter-based markers that dominate the field and could improve how treatment failure and therapeutic strategy are interpreted in areas of high malaria burden.

## Data availability

All the dataset utilized and generated in this manuscript have been deposited in https://zenodo.org/uploads/17010754. DOI: 10.5281/zenodo.17010754 ( Kiboi & Muriithi, 2025). An extra extended dataset utilised and generated during additional analysis for NADPH and NADP ^**+**^ docking, MD simulation, ligand (drug) docking and *PfFNR-PfFd* binding using haddock has been deposited in https://zenodo.org/records/20184463. DOI: 10.5281/zenodo.20184463 ( Kiboi & Muriithi, 2026).

## References

[ref1] Abraham M Alekseenko A Andrews B : GROMACS 2026.0 Manual. *Zenodo.* 2026. 10.5281/zenodo.17944198

[ref2] Abraham MJ Murtola T Schulz R : GROMACS: High performance molecular simulations through multi-level parallelism from laptops to supercomputers. *SoftwareX.* 2015;1-2:19–25. 10.1016/j.softx.2015.06.001

[ref3] Abramson J Adler J Dunger J : Accurate structure prediction of biomolecular interactions with AlphaFold 3. *Nature.* 2024;630(8016):493–500. 10.1038/s41586-024-07487-w 38718835 PMC11168924

[ref4] Akuh O-A Elahi R Prigge ST : The ferredoxin redox system – an essential electron distributing hub in the apicoplast of Apicomplexa. *Trends Parasitol.* 2022;38(10):868–881. 10.1016/j.pt.2022.08.002 35999149 PMC9481715

[ref5] Balconi E Pennati A Crobu D : The ferredoxin-NADP+ reductase/ferredoxin electron transfer system of Plasmodium falciparum. *Febs j.* 2009;276(14):3825–3836. 10.1111/j.1742-4658.2009.07100.x 19523113

[ref6] Balikagala B Fukuda N Ikeda M : Evidence of Artemisinin-Resistant Malaria in Africa. *N Engl J Med.* 2021;385(13):1163–1171. 10.1056/NEJMoa2101746 34551228

[ref7] Baroni S Pandini V Vanoni MA : A single tyrosine hydroxyl group almost entirely controls the NADPH specificity of Plasmodium falciparum ferredoxin-NADP+ reductase. *Biochemistry.* 2012;51(18):3819–3826. 10.1021/bi300078p 22519987

[ref8] Borges S Cravo P Creasey A : Genomewide scan reveals amplification of mdr1 as a common denominator of resistance to mefloquine, lumefantrine, and artemisinin in Plasmodium chabaudi malaria parasites. *Antimicrob Agents Chemother.* 2011;55(10):4858–4865. 10.1128/AAC.01748-10 21709099 PMC3186966

[ref9] ccsb-scripps : *AutoDock Vina (Version 1.2.7) [Computer software].*[GitHub.].2025. Reference Source

[ref10] Cichocki BA Donzel M Heimsch KC : Plasmodium falciparum Ferredoxin-NADP(+) Reductase-Catalyzed Redox Cycling of Plasmodione Generates Both Predicted Key Drug Metabolites: Implication for Antimalarial Drug Development. *ACS Infect Dis.* 2021;7(7):1996–2012. 10.1021/acsinfecdis.1c00054 33855850

[ref11] Cock PJA Antao T Chang JT : Biopython: freely available Python tools for computational molecular biology and bioinformatics. *Bioinformatics.* 2009;25(11):1422–1423. 10.1093/bioinformatics/btp163 19304878 PMC2682512

[ref12] Crobu D Canevari G Milani M : Plasmodium falciparum ferredoxin-NADP+ reductase His286 plays a dual role in NADP(H) binding and catalysis. *Biochemistry.* 2009;48(40):9525–9533. 10.1021/bi9013209 19736991

[ref13] Koning-Ward TF de Boddey JA Fowkes FJI : Molecular approaches to Malaria 2020. *Cell Microbiol.* 2021;23(3):e13289. 10.1111/cmi.13289 33197142

[ref14] Dominguez C Boelens R Bonvin AM : HADDOCK: a protein-protein docking approach based on biochemical or biophysical information. *J Am Chem Soc.* 2003;125(7):1731–1737. 10.1021/ja026939x 12580598

[ref15] Dondorp AM Nosten F Yi P : Artemisinin resistance in Plasmodium falciparum malaria. *N Engl J Med.* 2009;361(5):455–467. 10.1056/NEJMoa0808859 19641202 PMC3495232

[ref16] Eastman RT Fidock DA : Artemisinin-based combination therapies: a vital tool in efforts to eliminate malaria. *Nat Rev Microbiol.* 2009;7(12):864–874. 10.1038/nrmicro2239 19881520 PMC2901398

[ref17] Eberhardt J Santos-Martins D Tillack AF : AutoDock Vina 1.2.0: New Docking Methods, Expanded Force Field, and Python Bindings. *J Chem Inf Model.* 2021;61(8):3891–3898. 10.1021/acs.jcim.1c00203 34278794 PMC10683950

[ref18] Goodman CD McFadden GI : Targeting apicoplasts in malaria parasites. *Expert Opin Ther Targets.* 2013;17(2):167–177. 10.1517/14728222.2013.739158 23231425

[ref19] Halsey ES Plucinski MM : Out of Africa: Increasing reports of artemether-lumefantrine treatment failures of uncomplicated Plasmodium falciparum infection. *J Travel Med.* 2023;30(8). 10.1093/jtm/taad159 38109778 PMC10893888

[ref20] Harris CR Millman KJ Walt SJ van der : Array programming with NumPy. *Nature.* 2020;585(7825):357–362. 10.1038/s41586-020-2649-2 32939066 PMC7759461

[ref21] Honorato RV Trellet ME Jiménez-García B : The HADDOCK2.4 web server for integrative modeling of biomolecular complexes. *Nat Protoc.* 2024;19(11):3219–3241. 10.1038/s41596-024-01011-0 38886530

[ref22] Hunt P Afonso A Creasey A : Gene encoding a deubiquitinating enzyme is mutated in artesunate- and chloroquine-resistant rodent malaria parasites. *Mol Microbiol.* 2007;65(1):27–40. 10.1111/j.1365-2958.2007.05753.x 17581118 PMC1974797

[ref23] Kiboi D Bushell E Ndung’u L : Genome analysis of lumefantrine-resistant Plasmodium berghei reveals a new protein associated with multidrug resistance [Poster No: 67]. *Molecular Approaches to Malaria (MAM) Conference.* Lorne, Victoria, Australia;February 23–27, 2020.

[ref24] Kiboi D Irungu B Orwa J : Piperaquine and Lumefantrine resistance in Plasmodium berghei ANKA associated with increased expression of Ca2+/H+ antiporter and glutathione associated enzymes. *Exp Parasitol.* 2014;147:23–32. 10.1016/j.exppara.2014.10.008 25448357 PMC6128398

[ref25] Kiboi D Muriithi B : Extended data: Structural and Functional Impact of the G340S Mutation in Plasmodium falciparum Ferredoxin NADP ^+^ Reductase: In silico Analysis and Molecular Docking. 2025. 10.5281/zenodo.17010754

[ref26] Kiboi D Muriithi B : Extended data 2: Structural and Functional Impact of the G340S Mutation in Plasmodium falciparum Ferredoxin NADP ^+^ Reductase: In silico Analysis and Molecular Docking. 2026. 10.5281/zenodo.20217529

[ref27] Kiboi DM Irungu BN Langat B : Plasmodium berghei ANKA: selection of resistance to piperaquine and lumefantrine in a mouse model. *Exp Parasitol.* 2009;122(3):196–202. 10.1016/j.exppara.2009.03.010 19318094 PMC2691925

[ref28] Kimata-Ariga Y Chikuma Y Saitoh T : NADP(H) allosterically regulates the interaction between ferredoxin and ferredoxin-NADP(+) reductase. *FEBS Open Bio.* 2019;9(12):2126–2136. 10.1002/2211-5463.12752 31665566 PMC6886308

[ref29] Kimata-Ariga Y Morihisa R : Effect of Artemisinin on the Redox System of NADPH/FNR/Ferredoxin from Malaria Parasites. *Antioxidants (Basel).* 2022;11(2). 10.3390/antiox11020273 35204156 PMC8868210

[ref30] Kimata-Ariga Y Saitoh T Ikegami T : Molecular interaction of ferredoxin and ferredoxin-NADP+ reductase from human malaria parasite. *J Biochem.* 2007;142(6):715–720. 10.1093/jb/mvm184 17938142

[ref31] Lesanavičius M Aliverti A Šarlauskas J : Reactions of Plasmodium falciparum Ferredoxin:NADP(+) Oxidoreductase with Redox Cycling Xenobiotics: A Mechanistic Study. *Int J Mol Sci.* 2020;21(9). 10.3390/ijms21093234 32370303 PMC7247349

[ref32] Lopez MJ Mohiuddin SS : *Biochemistry, Essential Amino Acids.* StatPearls Publishing;2024. https://www.ncbi.nlm.nih.gov/books/NBK557845/ 32496725

[ref33] Mbye H Mane K Diop MF : Plasmodium falciparum merozoite invasion ligands, linked antimalarial resistance loci and ex vivo responses to antimalarials in The Gambia. *J Antimicrob Chemother.* 2022;77(11):2946–2955. 10.1093/jac/dkac244 35904009 PMC9616547

[ref34] Meng EC Goddard TD Pettersen EF : UCSF ChimeraX: Tools for structure building and analysis. *Protein Science.* 2023;32(11):e4792. 10.1002/pro.4792 37774136 PMC10588335

[ref35] Meng EC Pettersen EF Couch GS : Tools for integrated sequence-structure analysis with UCSF Chimera. *BMC Bioinformatics.* 2006;7:339. 10.1186/1471-2105-7-339 16836757 PMC1570152

[ref36] Milani M Balconi E Aliverti A : Ferredoxin-NADP+ reductase from Plasmodium falciparum undergoes NADP+-dependent dimerization and inactivation: functional and crystallographic analysis. *J Mol Biol.* 2007;367(2):501–513. 10.1016/j.jmb.2007.01.005 17258767

[ref37] Miotto O Amato R Ashley EA : Genetic architecture of artemisinin-resistant Plasmodium falciparum. *Nat Genet.* 2015;47(3):226–234. 10.1038/ng.3189 25599401 PMC4545236

[ref38] Mistry J Chuguransky S Williams L : Pfam: The protein families database in 2021. *Nucleic Acids Res.* 2020;49(D1):D412–D419. 10.1093/nar/gkaa913 33125078 PMC7779014

[ref39] Mungthin M Khositnithikul R Sitthichot N : Association between the pfmdr1 gene and in vitro artemether and lumefantrine sensitivity in Thai isolates of Plasmodium falciparum. *Am J Trop Med Hyg.* 2010;83(5):1005–1009. 10.4269/ajtmh.2010.10-0339 21036827 PMC2963959

[ref40] O’Boyle NM Banck M James CA : Open Babel: An open chemical toolbox. *J Cheminform.* 2011;3:33. 10.1186/1758-2946-3-33 21982300 PMC3198950

[ref41] Punta M Coggill PC Eberhardt RY : The Pfam protein families database. *Nucleic Acids Res.* 2011;40(D1):D290–D301. 10.1093/nar/gkr1065 22127870 PMC3245129

[ref42] Ralph SA Dooren GG van Waller RF : Tropical infectious diseases: metabolic maps and functions of the Plasmodium falciparum apicoplast. *Nat Rev Microbiol.* 2004;2(3):203–216. 10.1038/nrmicro843 15083156

[ref43] Rosenthal PJ Asua V Bailey JA : The emergence of artemisinin partial resistance in Africa: how do we respond? *Lancet Infect Dis.* 2024;24(9):e591–e600. 10.1016/S1473-3099(24)00141-5 38552654 PMC12954456

[ref44] Seeber F Soldati-Favre D : Chapter 5 - Metabolic Pathways in the Apicoplast of Apicomplexa.In Jeon KW , editor. *International Review of Cell and Molecular Biology.* Academic Press; Vol.281, pp.161–228;2010. 10.1016/S1937-6448(10)81005-6 20460186

[ref45] Sievers F Wilm A Dineen D : Fast, scalable generation of high-quality protein multiple sequence alignments using Clustal Omega. *Mol Syst Biol.* 2011;7(1):MSB201175. 10.1038/msb.2011.75 21988835 PMC3261699

[ref46] Sigrist CJA Castro E de Cerutti L : New and continuing developments at PROSITE. *Nucleic Acids Res.* 2012;41(D1):D344–D347. 10.1093/nar/gks1067 23161676 PMC3531220

[ref47] Sisowath C Strömberg J Mårtensson A : In vivo selection of Plasmodium falciparum pfmdr1 86N coding alleles by artemether-lumefantrine (Coartem). *J Infect Dis.* 2005;191(6):1014–1017. 10.1086/427997 15717281

[ref48] Swift RP Rajaram K Elahi R : Roles of Ferredoxin-Dependent Proteins in the Apicoplast of Plasmodium falciparum Parasites. *mBio.* 2022;13(1):e0302321–e0303021. 10.1128/mbio.03023-21 35164549 PMC8844926

[ref49] Trott O Olson AJ : AutoDock Vina: improving the speed and accuracy of docking with a new scoring function, efficient optimization, and multithreading. *J Comput Chem.* 2010;31(2):455–461. 10.1002/jcc.21334 19499576 PMC3041641

[ref50] Tumwebaze PK Conrad MD Okitwi M : Decreased susceptibility of Plasmodium falciparum to both dihydroartemisinin and lumefantrine in northern Uganda. *Nat Commun.* 2022;13(1):6353. 10.1038/s41467-022-33873-x 36289202 PMC9605985

[ref51] Zundert GCP van Rodrigues JPGLM Trellet M : The HADDOCK2.2 Web Server: User-Friendly Integrative Modeling of Biomolecular Complexes. *J Mol Biol. * 2016;428(4):720–725. 10.1016/j.jmb.2015.09.014 26410586

[ref52] Vanaerschot M Lucantoni L Li T : Hexahydroquinolines are antimalarial candidates with potent blood-stage and transmission-blocking activity. *Nat Microbiol.* 2017;2(10):1403–1414. 10.1038/s41564-017-0007-4 28808258 PMC5708124

[ref53] Vásquez-Vivar J Augusto O : Hydroxylated metabolites of the antimalarial drug primaquine. Oxidation and redox cycling. *J Biol Chem.* 1992;267(10):6848–6854. 10.1016/S0021-9258(19)50504-X 1313024

[ref54] Ward KE Fidock DA Bridgford JL : Plasmodium falciparum resistance to artemisinin-based combination therapies. *Curr Opin Microbiol.* 2022;69:102193. 10.1016/j.mib.2022.102193 36007459 PMC9847095

[ref55] WHO : *Strategy to respond to antimalarial drug resistance in Africa.* 2022. Reference Source

[ref56] WHO : *World malaria report 2025*.2025. Reference Source

